# Supplementary material for: Formation of topologically associated chromatin domains using quantum annealing
Source: Sci Rep. 2026 May 29;16:22952. doi: 10.1038/s41598-026-55306-1 (PMC13392026; doi:10.1038/s41598-026-55306-1)
Supplement: Supplementary file 1 — Supplementary Information. [file 41598_2026_55306_MOESM1_ESM.pdf]

Intermediate State Formation of Topologically Associated Chromatin Domains using Quantum Annealing: Supplementary Materials

Tobias Kempe,[1,*] S.M. Ali Tabei,[2,†] and Mohammad H. Ansari[3,‡]

[1]*Department of Physics, RWTH Aachen University, Aachen, Germany*
[2]*Department of Physics, University of Northern Iowa, Cedar Falls, Iowa 50614, United States*
[3]*Peter Grünberg Institute (PGI-12), Forschungszentrum Jülich, Jülich 52428, Germany*

[*] tkempe96@gmail.com
[†] tabei@uni.edu; Corresponding author
[‡] m.ansari@fz-juelich.de; Corresponding author

I. EMBEDDING ON D-WAVE TOPOLOGY

Based on the objective Hamiltonian, the required logical couplings between qubits and the equivalent objective graph are known [1, 2]. Before being able to perform this Hamiltonian on a quantum annealing sampling device, an embedding between the objective graph and the target graph of the device must be found which is in general non-trivial. The general structure of the objective graph is explained through figure **??** as the Cartesian product between a complete graph (clique) and a modified ring graph. Consequently, embedding strategies for these types of sub-graphs can help in the global embedding. For clique embedding, sophisticated strategies for all considered graph topologies (Chimera, Pegasus and Zephyr) are known (see [3] and [4]). Embeddings in this work are generated using **minorminer** code module from the D-Wave programming API.

Earlier-generation systems, such as the D-Wave 2000Q, utilized the Chimera topology, composed of sparsely connected unit cells implementing bipartite $K_{4,4}$ graphs. The newer Advantage platform adopts the Pegasus topology, significantly enhancing qubit connectivity (up to 15 couplers per qubit), thereby facilitating more efficient minor embeddings with reduced chain lengths and auxiliary overhead.

Although D-Wave systems do not incorporate full quantum error correction, they offer a scalable analog platform for investigating optimization and sampling problems within a quantum annealing paradigm [5, 6]. This makes them particularly useful for tasks that can be efficiently mapped onto the device's native sparse connectivity graph.

Considering the maximum model configuration $[12, 25, 5]$ with periodic boundary conditions for the selected target topologies, following optimal embedding characteristics emerge. Figure 3 shows the objective graph and associated embedding on the three considered topologies while table I lists the corresponding characteristics of the embedding. Embedding of the full model configuration is not possible on the Chimera graph of 2000Q. Specifically, a Chimera graph of size 42×42 is required to embed the maximum size model which is significantly larger than the graph of the D-Wave 2000Q device. This embedding shown in figure 3B results in a chain length of 32.33 on average and 62 at maximum which is to be expected to significantly hinder the Hamiltonian's ability to keep the qubit chains intact. The Pegasus topology is able to embed the maximum-size model on a graph size slightly smaller than that of the Advantage device and produces a maximum chain length of 21 which has a factor of 3 to the recommended limit. The Zephyr topology uses approximately $70\,\%$ of the graph available on the announced Advantage2 device while having a maximum chain length of 17. Even though Pegasus and Zephyr have higher than optimal chain lengths, the performance of their embeddings turns out acceptable. Since for the Zephyr topology only a prototype of size 4×4 is available at the time of writing, the Advantage device with the Pegasus topology is selected as the best option for sampling from large-scale models.

Topology	Chimera	Pegasus	Zephyr
Required Size	42×42	14×14	10×10
$\langle L_C \rangle$	32.33	11.67	9.17
$L_{C,\text{max}}$	62	21	17

TABLE I. Characteristics of different target topologies for the maximum size model $[12, 25, 5]$. The required size refers to the size of the Chimera, Pegasus and Zephyr graphs as defined in [7]. $\langle L_C \rangle$ and $L_{C,\text{max}}$ refer to the average and maximum chain lengths of the corresponding embedding on the minimally possible graph size.

II. PROCESSING OF EPIGENOMIC DATA

The epigenetic data used in this paper was gathered from the E017-IMR90 dataset of human fetal lung fibroblast cell genes recorded via the ChIP sequencing method. The respective strengths of all considered epigenetic markers were extracted as signal tracks across chromosome 9 The data was downloaded from the Roadmap Epigenomics Project at `https://egg2.wustl.edu/roadmap/web_portal/` (see [8]) in `.bigwig` format. It was compiled for further analysis by the `bigWigToBedGraph` executable, obtained from the binary utilities directory of UCSC: `http://hgdownload.soe.ucsc.edu/admin/exe/linux.x86_64/` (see [9]).

As an example, extracting the strengths of the H3K4me1 marker between base pairs $50'000$ and $100'000$ works like this (executed on a Linux or WSL system):

```
./bigWigToBedGraph E017-H3K4me1.pval.signal.bigwig H3K4me1.bedGraph -start=50000
-end=100000
```

III. SCALING BEHAVIOR

The feasibility of the proposed model depends on its scaling behavior for larger model sizes. Although pertinently larger models are not performable on current quantum annealing sampling devices, the scaling of the embedding can give valueable

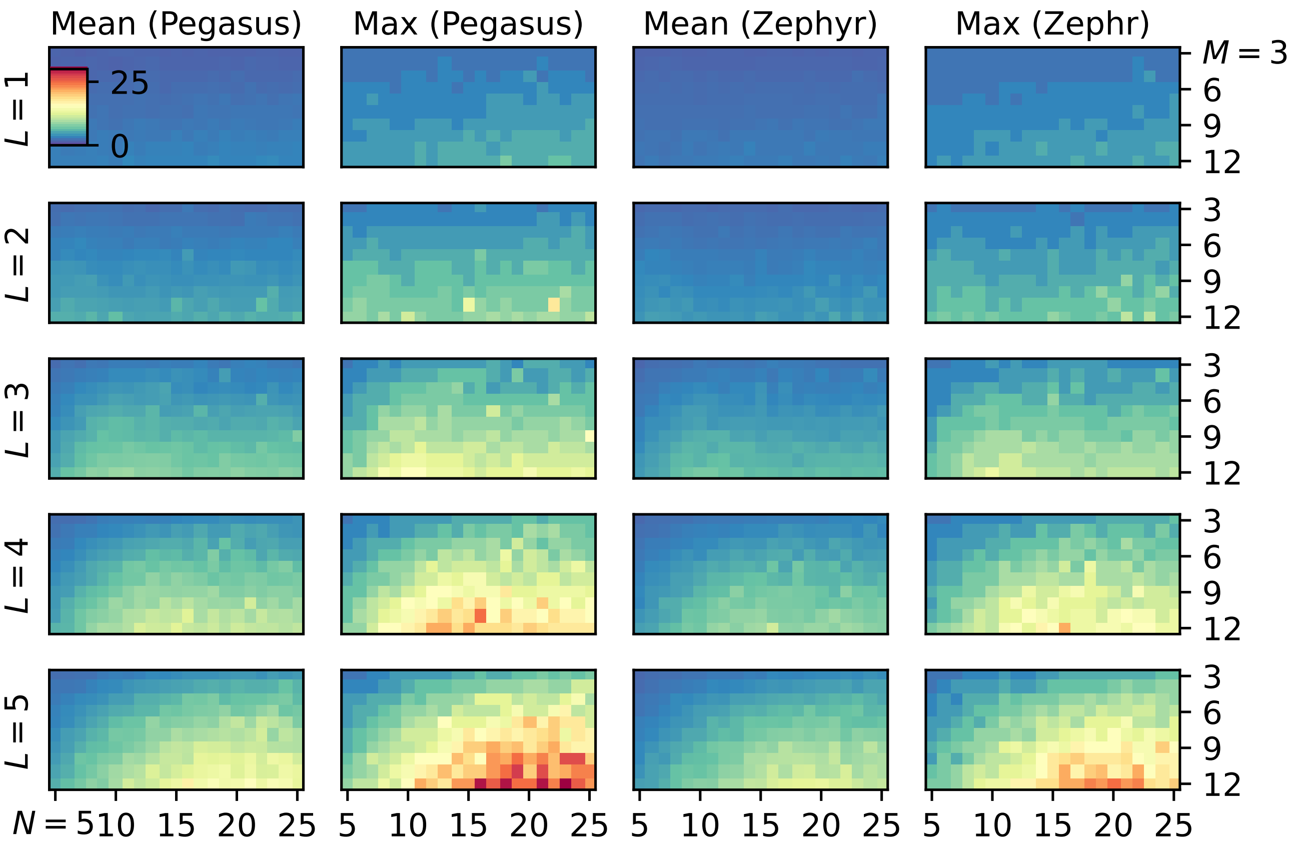

FIG. 1. Scaling behavior of the objective graph embedding on Pegasus and Zephyr topologies. Shown are average and maximum chain lengths of corresponding embeddings of all model sizes between $[3, 5, 1]$ and $[12, 25, 5]$.

insights as well. This is because on the one hand the embedding determines the required target size to embed an objective graph physically and on the other hand other works show that the embedding characteristics are a potent indicator for success of the sampling. As an example, D-Wave itself recommends a maximum chain length of 7. Figure 1 shows a grid sweep of all possible combinations of model sizes $[M, N, L]$ and the associated average and maximum chain lengths of the embedding. Since the algorithm for computing embeddings works non-deterministically using a heuristic [10], there is a certain variance in the displayed data. A representative amount of repetitions to gain accurate mean values was not done due to long computation times for large model sizes; however, such examinations for model sizes up to $[5, 12, 3]$ reveal a non-growing standard deviation of ~ 0.27 for the average and ~ 0.71 for the maximum chain length in 300 samples. Consequently a similar standard deviation is assumed for larger model sizes, as well. As expected, the scaling for medium-scale models in figure 2 shows that the maximum chain length does not scale with the node-length of the model. It rather scales with the correlation length and with the number of considered epigenetic marks. As expected and as also observed in other applications, the Pegasus topology requires larger chains than the Zephyr topology.

IV. BOUNDARY CONDITIONS

Boundary conditions are used for the inter-nucleosomic coupling parameters of the model to improve the sampling performance and to make sampling results independent of the nucleosomic grid location (in analogy to [11]). Nevertheless, introducing additional couplings - especially those with otherwise great qubit distance - increases the complexity of the embedding. A comparison between open and periodic boundary conditions is shown in figure 4. The embeddings of open models are almost strictly smaller than their periodic counterparts. For varying M the difference is almost negligible and for N depends strongly on the exact microscopic fit between the objective and target graphs, which results in jumping embedding sizes. The gap between open and periodic boundary conditions increases for growing L as the width of the periodic graph loop gets wider.

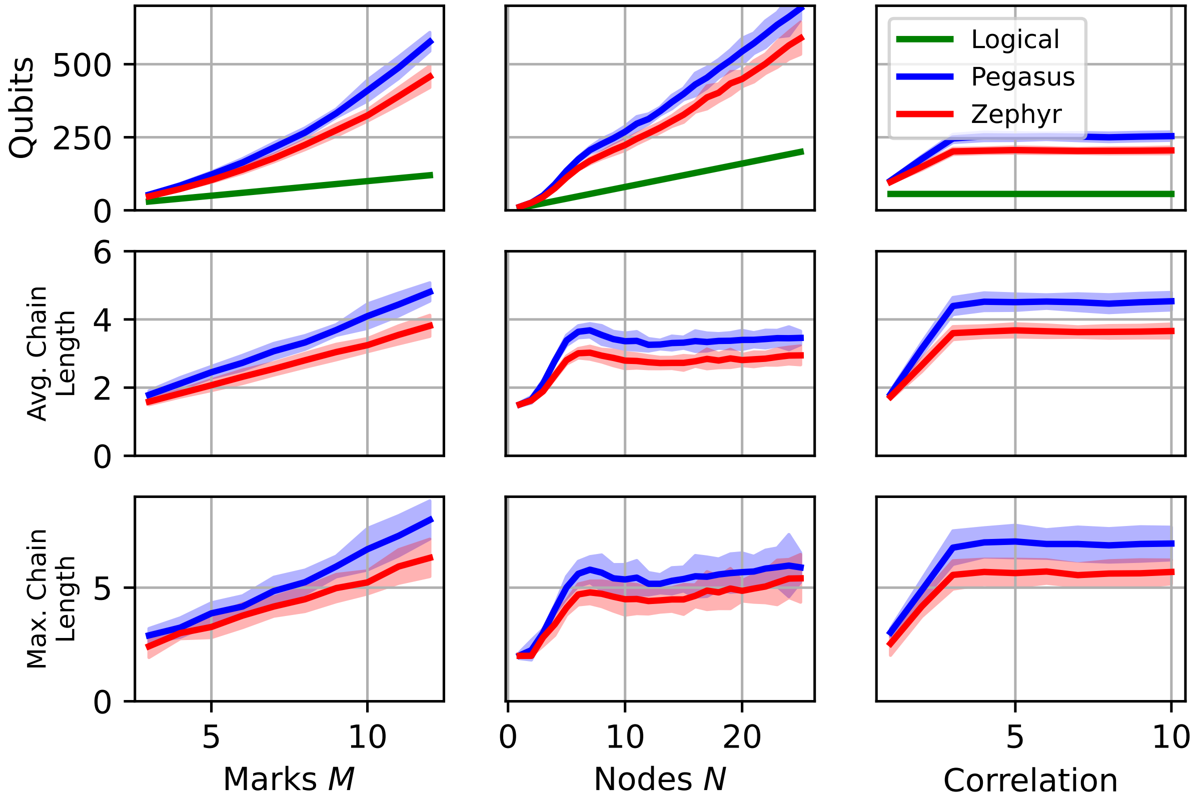

FIG. 2. Scaling behavior regarding number of nucleosomes, epigenetic marks and maximum coupling length for medium-scale models.

V. COUPLING THRESHOLD

Besides the size of the model itself, the embedding complexity can be influenced by compressing the model parameters. This refers to the omission of couplings that have an absolute coupling strength below a certain threshold $\delta > \max(|J_i|)$. It is important to note, that the threshold must be applied to the coupling parameters in Ising formulation, not in QUBO formulation. This is crucial, since the Ising formulation corresponds to the physical qubit detunings and interactions. This is approximately equivalent to the exact model, since non-existent couplings have the same effect as couplings with strength zero [12]. This approach is especially sensible for the distribution of QUBO parameters that show a high frequency in vicinity of zero (refer to figure **??**).[13] The effect of a coupling threshold is shown in figure 5 and indicates that the average chain length decreases approximately linearly towards 1.

VI. PLOTS

[1] X. Xu, Manabputra, C. Vignes, M. H. Ansari, and J. M. Martinis, "Lattice hamiltonians and stray interactions within quantum processors," *Physical Review Applied*, vol. 22, no. 6, p. 064030, 2024.

[2] J. Ku, X. Xu, M. Brink, D. C. McKay, J. B. Hertzberg, M. H. Ansari, and B. Plourde, "Suppression of unwanted zz interactions in a hybrid two-qubit system," *Physical review letters*, vol. 125, no. 20, p. 200504, 2020.

[3] K. Boothby, A. D. King, and A. Roy, "Fast clique minor generation in chimera qubit connectivity graphs," 2015.

[4] C. Klymko, B. D. Sullivan, and T. S. Humble, "Adiabatic quantum programming: Minor embedding with hard faults," 2012.

[5] A. Rajak, S. Suzuki, A. Dutta, and B. K. Chakrabarti, "Quantum annealing: An overview," *Philosophical Transactions of the Royal Society A*, vol. 381, no. 2241, p. 20210417, 2023.

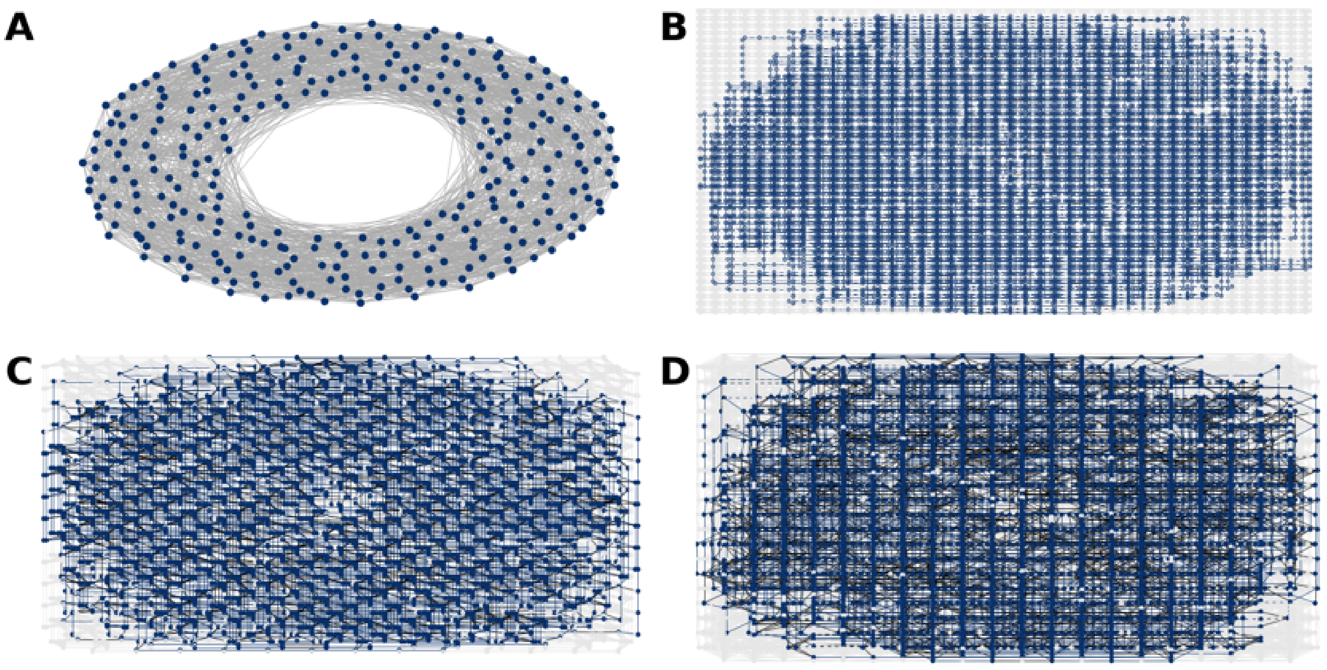

FIG. 3. Embedding of full-scale model on all three considered topologies. (A) Full-scale model ($[12, 25, 5]$) with periodic boundary conditions. (B) Embedding on 42×42 Chimera topology. (C) Embedding on 14×14 Pegasus topology. (D) Embedding on 10×10 Zephyr topology.

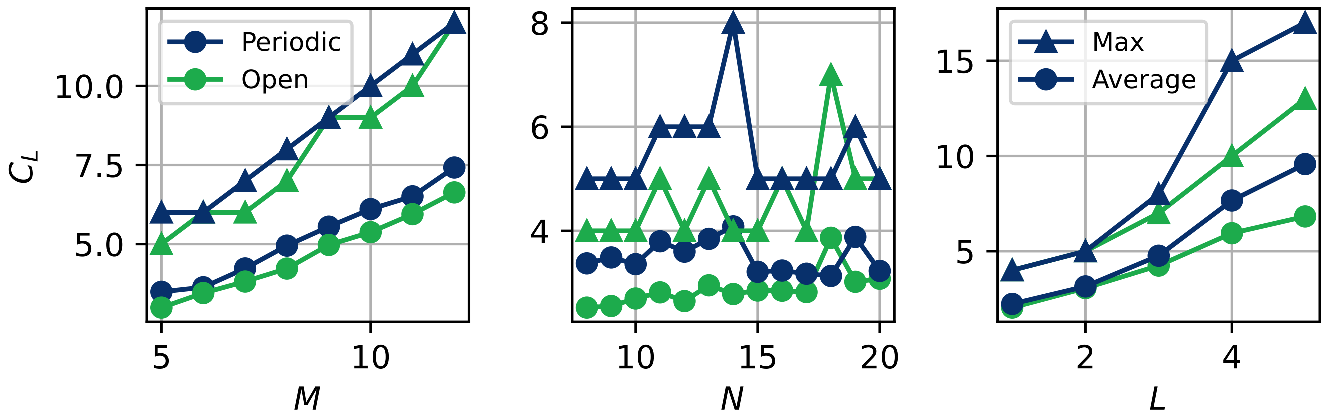

FIG. 4. Comparison of chain lengths for models with open vs. periodic boundary conditions with varying model parameters M, N and L.

[6] J. Pazem and M. H. Ansari, "Error mitigation in brainbox quantum autoencoders," *Scientific Reports*, vol. 15, no. 1, p. 2257, 2025.

[7] Kelly Boothby and Paul Bunyk and Jack Raymond and Aidan Roy, "Next-Generation Topology of D-Wave Quantum Processors," tech. rep., D-Wave Systems Inc., 3033 Beta Ave, Burnaby, BC V5G 4M9, Canada, Feb. 2020.

[8] NIH Roadmap Epigenomics Mapping Consortium, "Roadmap epigenomics project." `https://egg2.wustl.edu/roadmap/web_portal/`. Accessed: 2023-01-10.

[9] University of California Santa Cruz Genomics Institute, "Binary utilities directory." `http://hgdownload.soe.ucsc.edu/admin/exe/linux.x86_64/`. Accessed: 2023-03-04.

[10] J. Cai, W. G. Macready, and A. Roy, "A practical heuristic for finding graph minors," 2014.

[11] W. J. Xie and B. Zhang, "Learning the formation mechanism of domain-level chromatin states with epigenomics data," *Biophysical Journal*, vol. 116, no. 10, pp. 2047–2056, 2019.

[12] M. H. Ansari, F. K. Wilhelm, U. Sinha, and A. Sinha, "The effect of environmental coupling on tunneling of quasiparticles in josephson junctions," *Superconductor Science and Technology*, vol. 26, no. 12, p. 125013, 2013.

[13] The argument also holds for Ising parameters, since the couplings are only scaled with a constant factor during the conversion.

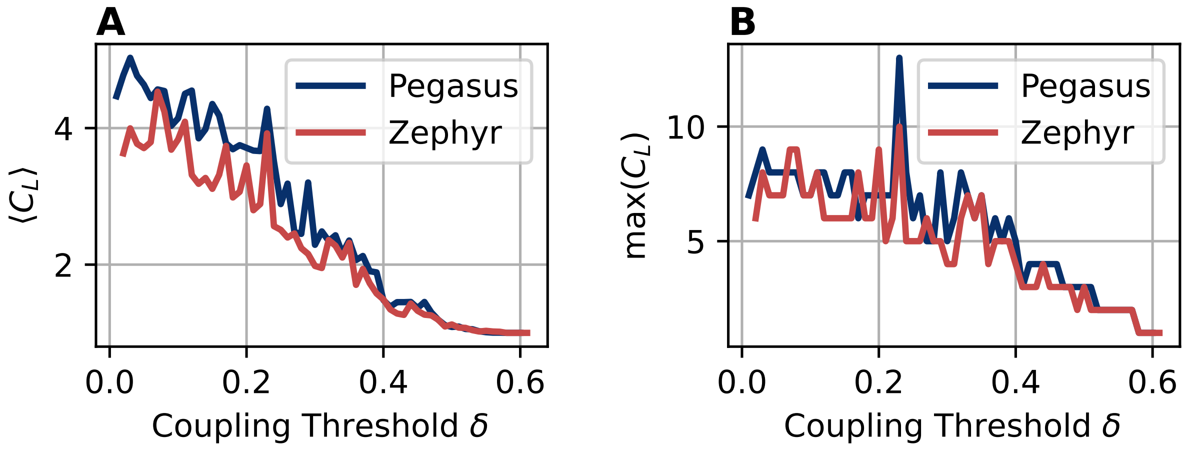

FIG. 5. Average and maximum chain strength dependent on coupling threshold δ in embedding on Pegasus and Zephyr target graphs.

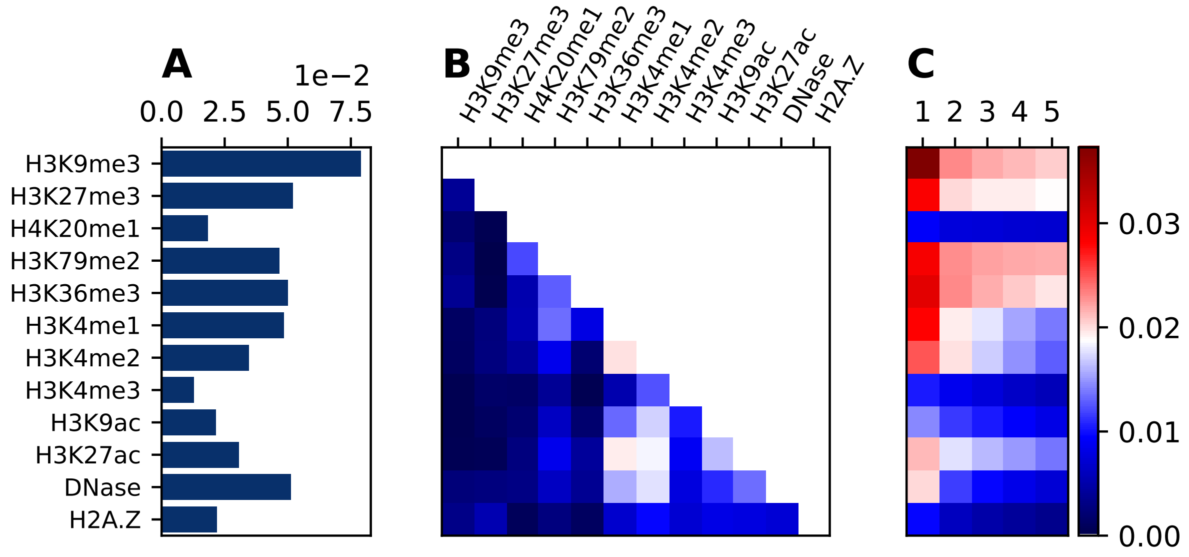

FIG. 6. Statistics of the entire binarized dataset of 140 million base pairs or 700.000 boolean incidence bins, respectively, per epigenetic mark. (A) Mean incidence of each epigenetic mark on the entirety of the dataset. (B) Intra-nucleosome correlation between any two epigenetic marks on the same nucleosome. (C) Inter-nucleosome correlation between the incidence of an epigenetic mark and the incidence of the same mark on neighboring nucleosome with distance $l \in [1 \cdots L]$ where $L = 5$.

[14] D-Wave Systems Inc., "Qpu-specific characteristics." https://docs.dwavesys.com/docs/latest/doc_physical_properties.html, 2023. Accessed: 2023-04-26.

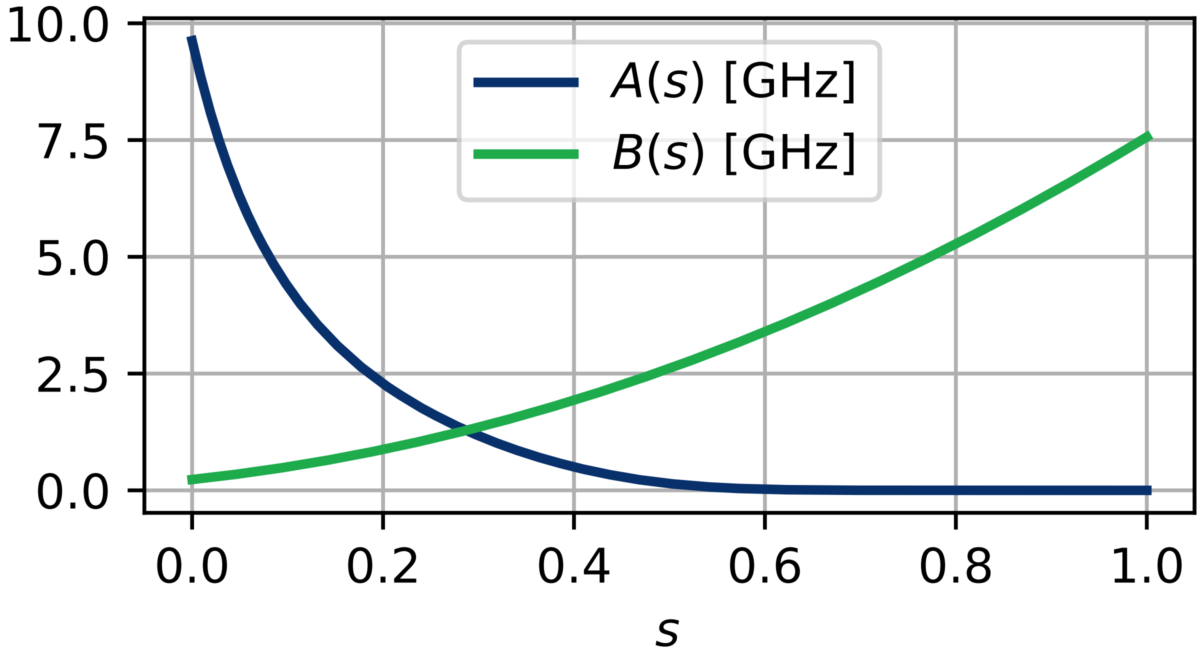

FIG. 7. Default annealing functions $A(s)$ and $B(s)$ for the D-Wave Advantage machine. [14]
